# Supplementary figures and images for: A unified mechanism for the control of Drosophila wing growth by the morphogens Decapentaplegic and Wingless
Source: PLoS Biol. 2021 Mar 3;19(3):e3001111. doi: 10.1371/journal.pbio.3001111 (PMC8148325; doi:10.1371/journal.pbio.3001111)

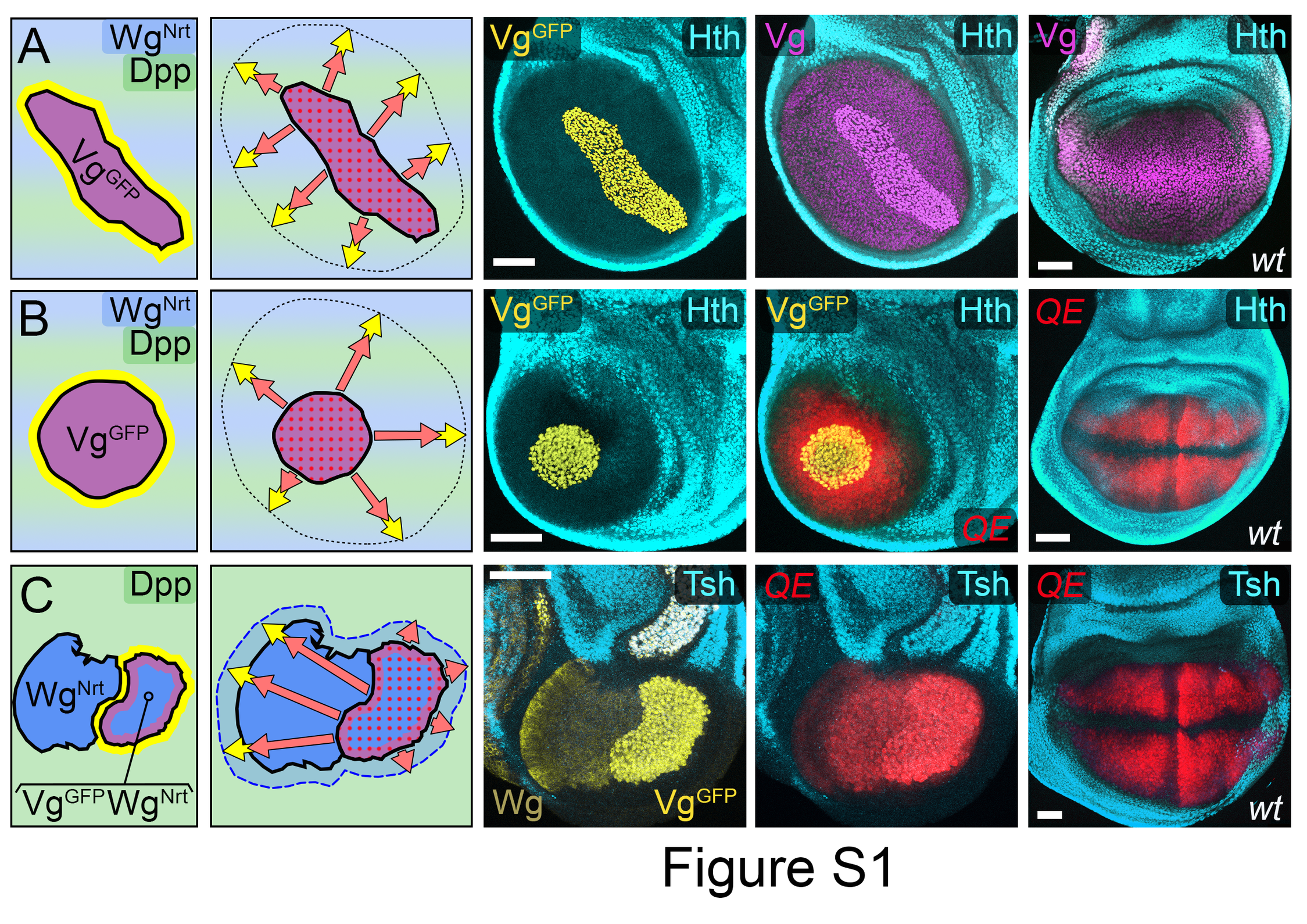

Supplement: S1 Fig — (A, B) Tub>vgGFP clones (marked by GFP, bright yellow in the micrographs) in dppd apo UAS.dpp UAS>wgNrt discs monitored for either Vg (purple, A) or the 5XQE.DsRed reporter (red, B), as well as Hth (turquoise, A and B), a transcription factor that is expressed in prospective hinge and body wall territories surrounding the pre-wing domain (as shown for the corresponding wild-type discs to the right). The clones have induced FF growth in the surround (indicated by the halos of Vg and 5XQE.DsRed expressing cells); however, this is restricted to the pre-wing domain delimited by the surrounding Hth expressing hinge and body wall territories. (C) A dppd apo UAS.dpp disc containing a Tub>vgGFP UAS>wgNrt clone (bright yellow) next to a UAS>wgNrt (dim yellow) monitored for Wg, VgGFP, and 5XQE.DsRed (labeled as in B), as well as the transcription factor Tsh (turquois), which like Hth, demarcates the proximal hinge and body wall territories of the disc (as in the wild-type disc to the right). The Tub>vgGFP UAS>wgNrt clone expresses the 5XQE.DsRed reporter and has induced abutting wild-type cells to do the same (confirming that this clone is UAS>wgNrt in addition to Tub>vgGFP). This clone has also induced extensive FF growth throughout the abutting UAS>wgNrt clone and its immediate wild-type neighbors (monitored by 5XQE.DsRed expression). As in (A, B), all of the FF growth has occurred within the pre-wing region of the disc surrounded by the TshON HthON hinge and body wall territories. Key genotypes: dppd apo Tub>stop>vgGFP C765.Gal4 discs that are also UAS.dpp UAS>wgNrt (A, B), or that are UAS.dpp UAS>stop>wgNrt (C). Exact genotypes: A, B y w hsp70.flp/y w hsp70.flp; dppd8 ap56f UAS>wgNrt/dppd10 ap56f; Tubα1>CD2,y2>vgGFP C765.Gal4/UAS.dpp. C y w hsp70.flp/y w hsp70.flp 5XQE.DsRed; dppd8 ap56f UAS>CD2,y+>wgNrt/dppd10 ap56f; Tubα1>CD2,y2>vgGFP C765.Gal4/UAS.dpp. Dpp, Decapentaplegic; FF, feed-forward; FRT, >, Flp recombinase targets; Hth, Homothorax; QE, Quadrant enhancer; Tsh, [file pbio.3001111.s001.tif]

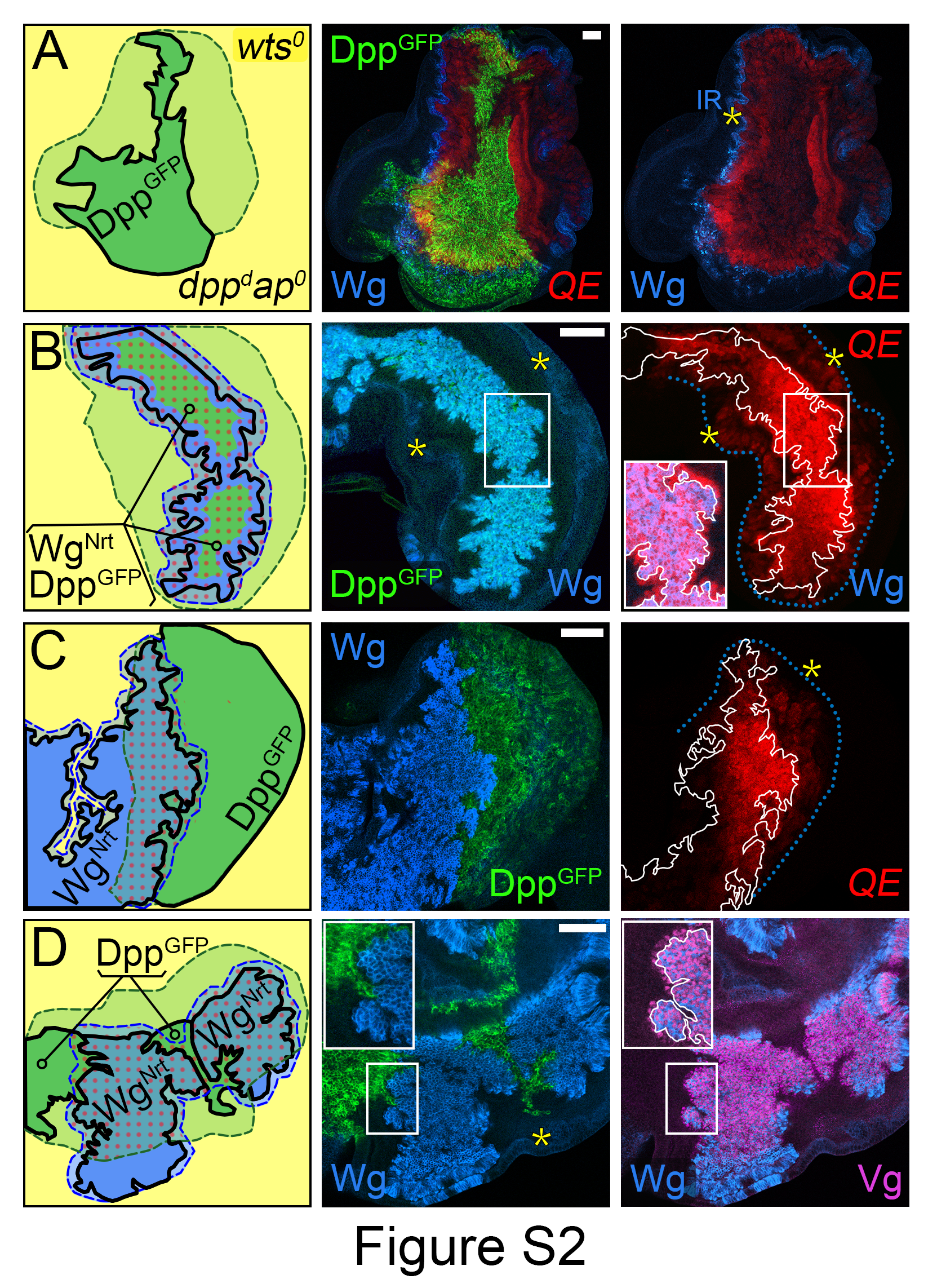

Supplement: S2 Fig — (A–D) dppd apo wtso discs carrying either UAS>dppGFP (A, green), UAS>wgNrt UAS>dppGFP (B, green/blue) or abutting UAS>dppGFP and UAS>wgNrt clones (C, D, green and blue, respectively), monitored for 5XQE.DsRed expression (A–C, red) or native Vg (D, purple). Uniform activity of the FF transduction pathway is indicated by yellow wash in the diagrams to the left; the Wg IR is indicated by yellow asterisks (A–D) and a dotted blue line (B, C) in the micrographs to the right. In dppd apo wtso discs, as in dppd fto apo discs (Fig 9A), 5XQE.DsRed activity is not detected and no FF growth occurs, even though the FF transduction pathway is constitutively active. (A) UAS>dppGFP clones generated in this background restore weakly detectable QE activity (red) and an expansion of the pre-wing territory, likely dependent on cryptic Wg expressed by IR cells in the prospective hinge (as in Figs 9B and 10D; note that the level of 5XQE.DsRed is elevated modestly in cells neighboring the IR). (B) UAS>dppGFP UAS>wgNrt clones (outlined in white) restore peak QE-dependent activity within the clone and abutting cells outside the clone and grow as wing tissue. (C, D) Abutting UAS>dppGFP and UAS>wgNrt clones in dppd apo wtso discs. As in dppd fto apo discs (Fig 9F and 9G), cells within the UAS>wgNrt clone (outlined in white in C and in the inset in D), as well as their immediate neighbors, express peak levels of both 5XQE.DsRed (C) and native Vg (D) and grow as wing tissue, provided that they are located close enough to the abutting UAS>dppGFP clones to receive Dpp. Key genotypes: dppd apo wtso C765.Gal4 UAS>stop>dppGFP UAS>stop>wgNrt discs (A–D all have UAS> dppGFP clones, whereas B, C, but not A, have UAS>wgNrt clones). Exact genotypes: A–C y w hsp70.flp/y w hsp70.flp 5XQE.DsRed; dppd8 ap56f UAS>CD2,y+>wgNrt/dppd10 ap56f; wtsP2 UAS>CD2,y+>dppGFP/wtsP2 C765.Gal4 D y w hsp70.flp/y w hsp70.flp; dppd8 ap56f UAS>CD2,y+>wgNrt/dppd10 ap56f; wtsP2 UAS>CD2,y+>dppGFP/wtsP2 C765.Gal4. ap, apterous; Dpp [file pbio.3001111.s002.tif]

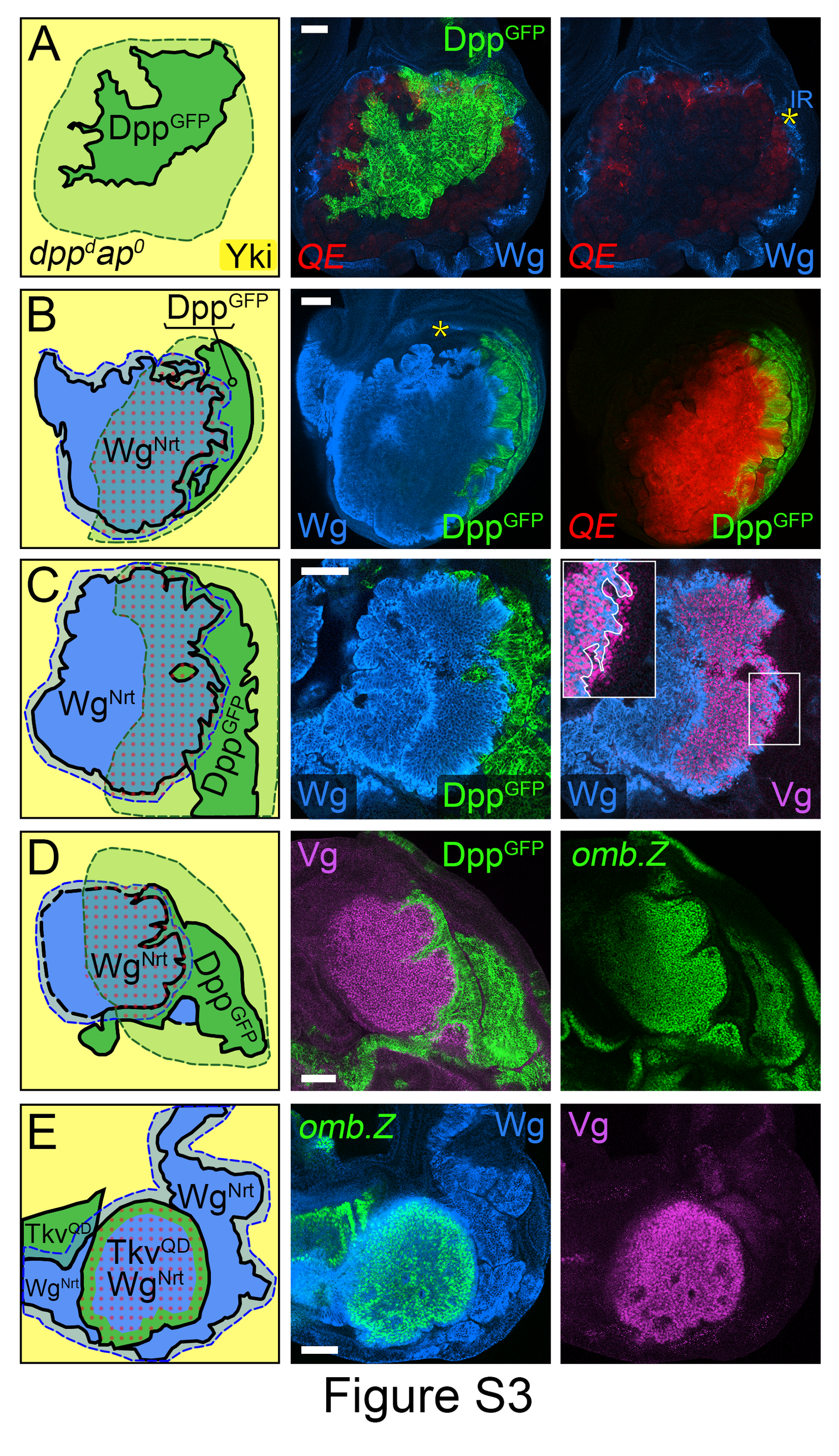

Supplement: S3 Fig — (A–D) dppd apo UAS.yki discs carrying either a single UAS>dppGFP clone (A), or abutting UAS>wgNrt and UAS>dppGFP clones (B–D), assayed for 5XQE.DsRed (red, A and B) or native Vg expression (purple, C and D). UAS>dppGFP clones are visualized by native fluorescence of the GFP tag (green) while the UAS>wgNrt clones are marked either directly (B, C) by WgNrt expression (blue) or indirectly (D) through their ability to induce peak Vg levels (purple) autonomously and at short-range (as in C). (A) UAS>dppGFP clones in dppd apo UAS.yki discs restore barely detectable QE activity and an expansion of the pre-wing territory (as in S2A Fig, 5XQE.DsRed expression is weakly elevated in pre-wing cells neighboring the surrounding Wg IR, consistent with IR cells being the source of cryptic Wg responsible for the QE response). (B–D) In contrast, UAS>dppGFP clones induce high-level QE-dependent Vg expression and extensive growth of neighboring UAS>wgNrt clones and their immediate neighbors, provided that the cells are close enough to receive Dpp (confirmed in D by expression of the omb.lacZ reporter; green, right panel; the UAS>wgNrt clone border is indicated by a white line in the inset in (C). (E) A dppd apo UAS.yki disc carrying UAS>tkvQD clones (marked by induction of omb.lacZ expression, green), UAS>wgNrt clones (marked by WgNrt expression, blue) and UAS>tkvQD UAS>wgNrt clones (green/blue). Vg expression (purple) is strictly autonomous to the UAS>tkvQD UAS>wgNrt clones. Key genotypes: dppd apo UAS.yki C765.Gal4 UAS>stop>wgNrt discs with either UAS>stop>dppGFP (A–D) or UAS>stop>tkvQD (E). Exact genotypes: A, B y w hsp70.flp/y w 5XQE.DsRed; dppd8 ap56f/dppd10 ap56f; UAS>CD2,y+>dppGFP UAS.yki; UAS>CD2,y+>wgNrt C765.Gal4. C, D y w hsp70.flp/y w omb.lacZ; dppd8 ap56f/dppd10 ap56f; UAS>CD2,y+>dppGFP UAS.yki; UAS>CD2,y+>wgNrt C765.Gal4. E y w hsp70.flp/y w omb.lacZ; dppd8 ap56f UAS>CD2,y+>wgNrt/dppd10 FRT39 ap56f; 2xUAS>CD2,y+>tkvQD C765.Gal4/UAS.yki. ap, apterous; Dpp, Decapentaplegic; [file pbio.3001111.s003.tif]

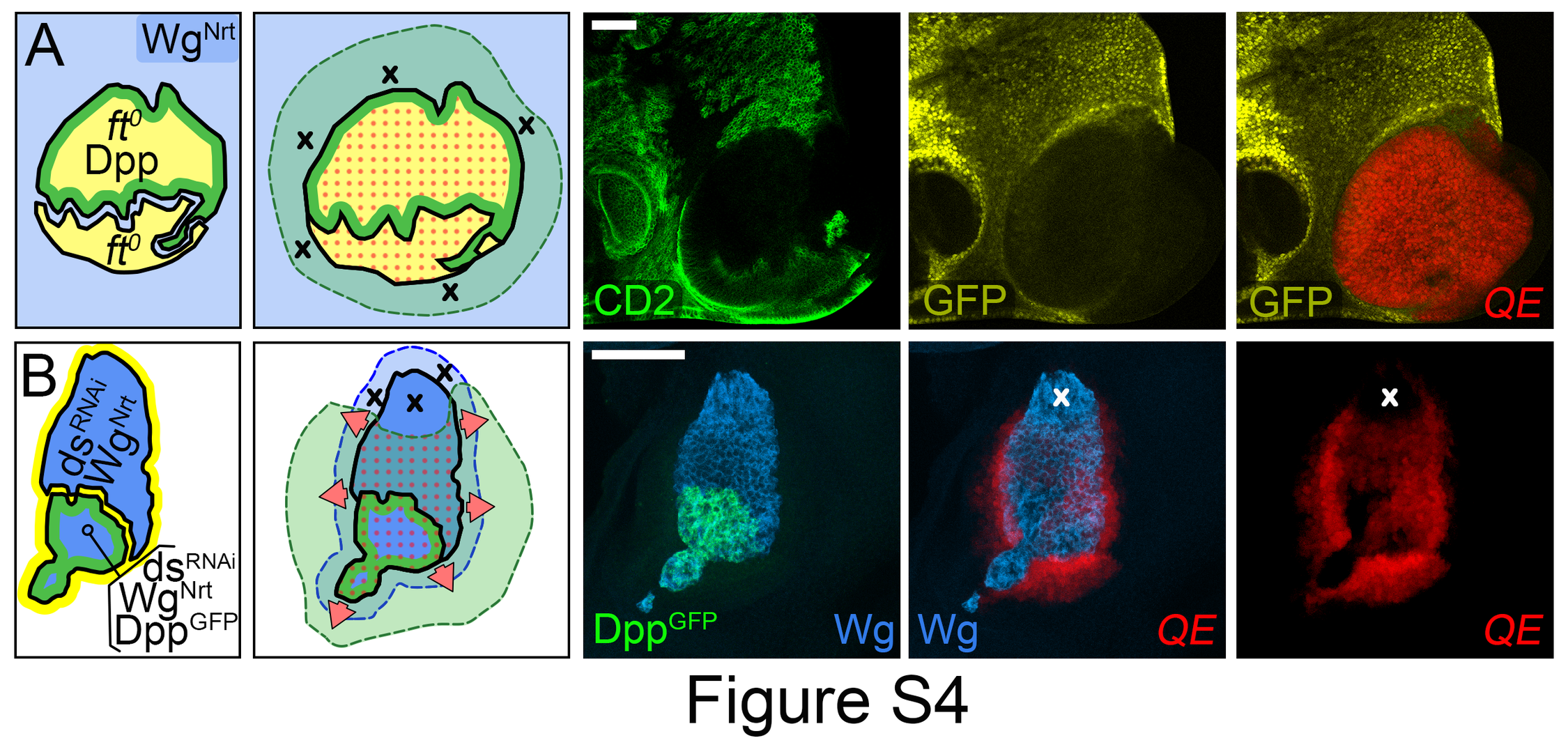

Supplement: S4 Fig — (A) A dppd apo disc with uniform WgNrt expression that contains 2 large clones both of which are mutant for ft (yellow in the cartoon; marked “black” by the loss of an hsp70.GFP marker, yellow, in the middle and right most micrographs), and one of which ectopically expresses Dpp (outlined in green; and marked “black” by the loss of CD2, green, resulting from excision of the stop cassette in UAS>CD2,stop>dpp). Cells within both clones grow autonomously as 5XQE.DsRed expressing wing tissue. Importantly, QE activation is strictly confined to the ft0 clones, indicating that vg-expressing wing cells that lack Ft cannot induce FF growth of abutting wild-type cells (X’s in the cartoon), even when the latter receive both Dpp and WgNrt (a third ft0 clone located to the left of these 2 ft0 clones fails to express 5XQE.DsRed because it is located outside of the pre-wing territory). (B) A dppd apo disc containing 2 UAS>wgNrt UAS.dsRNAi clones (blue, marked by WgNrt expression), one of which expresses DppGFP (green). Cells within these clones, as well as surrounding cells that can receive the tethered WgNrt signal, express 5XQE.DsRed and grow as wing tissue, provided that they are positioned close enough to the DppGFP expressing clone (cells that we infer are out of range are indicated by X’s). Thus, loss of either ft or ds activity cell-autonomously activates QE-dependent Vg expression in dppd apo discs that are supplied with exogenous Dpp and Wg, but only clones that have lost ds activity can induce FF growth of the surround, whereas those that have lost ft activity cannot, indicating that Ft itself is the vg-dependent, vg-inducing FF signal. Key genotypes: (A) dppd apo UAS>wgNrt UAS>CD2,stop>dpp C765.Gal4 disc that is also ft0 FRT39/hsp70.GFP FRT39. (B) dppd apo UAS.dsRNAi UAS>wgNrt UAS>CD2,stop>dppGFP Tub>Gal80,stop>Gal4 disc. Exact genotypes: A y w hsp70.flp/y w hsp70.flp 5XQE.DsRed; dppd8 ft15 FRT39 ap56f/dppd10 Dp(ft+) hsp70.GFPHA FRT39 ap56f; UAS>CD2,y+>dpp UAS>wgNrt/C76 [file pbio.3001111.s004.tif]
