# Supplementary material for: A unified mechanism for the control of Drosophila wing growth by the morphogens Decapentaplegic and Wingless
Source: PLoS Biol. 2021 Mar 3;19(3):e3001111. doi: 10.1371/journal.pbio.3001111 (PMC8148325; doi:10.1371/journal.pbio.3001111)
Supplement: Attachment — Submitted filename: PBIOLOGY-D-20-01965R1 Response to reviewers.docx [file pbio.3001111.s005.docx]

PBIOLOGY-D-20-01965R1 Response to Reviewers

Overview

All three reviewers agree that our findings and conclusions are comprehensive and compelling. Reviewers #1 and 2 found the paper to represent a significant advance. Reviewer #1 had no criticisms, but recommended acceptance as is. Reviewer #2 raised minor issues that we have addressed by revisions, or in a few cases in our responses below. Reviewer #3 had no substantive criticisms, but had reservations about whether our findings break new ground; we address these reservations below. Finally, we have made additional changes to improve the presentation of our experiments, interpretations and discussion in light of the reviewers’ critiques.

Below, we present a point-by-point response to the reviewers' comments, and indicate the changes made to the manuscript. Our responses are presented in blue font inserted into reviewers’ comments. Revisions we have made to address specific points are in red font.

We are aware that the complex genetic approaches we use in our experiments make for a challenging manuscript and thank the reviewers for taking the time and effort to provide thoughtful and informed comments.

Reviewers’ comments

Rev. 1:
In this manuscript Zecca and Struhl analyze the contribution of Wg, Dpp and Fat/Ds signaling to regulate vg expression and consequently the generation and expansion of the wing anlage of the Drosophila wing imaginal disc. They generate clones of cells expression a variety of genes in double mutant "ap dpp" wing discs, a genotype that precludes the generation of the wing primordium. In these discs they could unambiguously determine the individual and combined requirements for Dpp, Wg and Fat/Ds by providing the expression of these genes through the excision of transcriptional stop cassettes. In these discs they monitored the expression of several reporter genes, and use the non-autonomous expression of the vg quadrant enhancer as indication of the presence of wing tissue specified by induced vg expression. The experimental design is extremely sophisticated, but the authors have made a great effort to present their results in a way that could be followed by readers not familiar with the intricacies of clonal analysis and wing disc development. The manuscript is written in a way that the experimental and conceptual logic can be followed effortless, and the extensive use of colorful drawings and consistent color codes really help to visualize and understand the results. I understand that part of the analysis of conclusions related to Wg were already published, but I believe that putting together the results for Wg, Dpp and Wg+Dpp makes all sense and contributes to clarify the combined contribution of these pathways to wing growth. I can only congratulate the authors for yet another excellent piece of work and are in favor of publishing this manuscript as it is.

This reviewer found our presentation, experiments, findings and conclusions to be compelling and to represent a significant advance that merits publication “as is”.

Rev. 2:
Zecca and Struhl investigate growth control in the wing imaginal disc of Drosophila, specifically how the morphogens Wingless (Wg) and Decapentaplegic (Dpp) function together with the protocadherins Fat (Ft) and Dachsous (Ds) regulate this. Here they extend their previous studies, integrating Dpp into their 'feedforward' model. The model explains how Wg and Dpp can expand the size of the wing primordium in collaboration with the protocadherins Fat, so its size is limited by the distance to which these morphogens can move within the the wing disc. In addition, it is shown that both morphogens are continually required within the wing to promote proliferation. The simple proposal is that proliferation just requires a low threshold of both Wg and Dpp and above that the rate is constant. This is a much more attractive model that others that have been proposed, not least because it can easily explain why proliferation is fairly uniform throughout the developing wing pouch.

Agree!

A wealth of data is presented to support the model, most of it elegant and well explained. The figure legends are detailed and although there is some duplication with the main text this is very useful as the figures are quite complicated. The cartoons provided adjacent the images are also very helpful.

Agree!

The discussion is generally pertinent, although some issues are still not completely explained away, such as:

whether there are differences in growth control between lateral and medial regions of the wing . . .

We assume the reviewer is referring to the many previous findings that ectopic Dpp expression causes dramatic overgrowth of lateral portions of the wing primodium, but relatively little if any extra growth of medial portions.

To address this concern, we have revised the Discussion (page 42, paragraph 2) to clarify how the FF hypothesis accounts for this difference. As we explain in the revised text, we think this is because “medial” wing cells are located “behind” the FF recruitment interface and hence already receive levels of Dpp that exceed the minimum threshold required to sustain growth. Accordingly, adding extra Dpp to these cells should not cause additional growth. In contrast, “lateral” correspond to pre-wing cells located in “front” of the interface far from the source of Dpp. Hence, their ability to grow and be recruited into the wing is limited by the availability of Dpp. Providing these cells with ectopic Dpp overcomes this limitation, resulting in excess growth and recruitment, limited now only by the rate of propagation of the FF signal.

and in particular why membrane tethered Wg can drive such high levels of growth.

We assume the reviewer is referring to the previous and striking results of Alexandre et al [1], in which a knock-in allele of Wg that expresses a membrane tethered form of Wg in place of native Wg can nevertheless afford substantial rescue of normal wing growth.

This result has been viewed as evidence that Wg does not normally need to spread away from Wg expressing cells to exert a long-range influence on wing growth. Taken at face value, this interpretation presents a challenge to the FF hypothesis, which posits that it is the long-range, outward spread of Wg that drives wing growth. However, as we and others have noted, e.g, [2,3], there are many explanations of this result that do not contradict a normal requirement for long-range signaling activity of Wg. Indeed, as we emphasize in the revised Discussion, a requirement for such long-range action is already well established from experiments we published as long ago as 1996 [4] and reinforced by many subsequent studies (including our previous work [5–7], especially Fig. 3E in [6], and corroborated by our present findings). We make these points explicitly in the revised Discussion (page 44; paragraphs 1,2).

The latter issue has probably hampered acceptance of the author's previous studies, but now with the addition of Dpp into all this, with Dpp generally being accepted and a moveable morphogen in the wing, the model presented should gain wider appeal.

We agree that the uncritical interpretation of the results in the Alexendre et al paper has hampered acceptance of the FF model for the control of wing growth by Wg. However, as this reviewer argues at the end of his review, “*but now with the addition of Dpp into all this, with Dpp generally being accepted and a moveable morphogen in the wing, the model presented should gain wider appeal.*

This is a significant paper and all previous work should be reinterpreted in light of the data presented here.

Agree!

Other comments
The number of figures, however, cannot be ignored…are they all necessary? Most appear essential, but the authors could go through them again and decide what could be moved to Supplementary Data. Some suggestions [for moving figures to Supplementary Data]: Dpp morpho trap experiments (the tkvQ is a better reagent), all the Fig. 10 experiments

If requested as a condition of publication, we will move Figure 10 to Supplemental data. However, we view this (as well as the Dpp morphogen trap experiments, Figs. 4B,C) as key experimental contributions and hope our arguments below make a persuasive case that they should remain in the paper proper.

With respect to Figure 10: As we explain in the Results section of the revised manuscript, the experiments in Fig. 10 address and resolve a major challenge to the FF model (pages 32, paragraph 3). This is that FF model posits that both the induction and maintenance of QE-dependent Vg expression is dependent, in absolute terms, on the receipt of Wg, Dpp and the vg-dependent vg-inducing FF signal. Yet, *ap* null discs are devoid of D/V border cells — the source of Wg posited by the model — but can still be induced to express vg under QE control by ectopic Yki/Vg plus Dpp. At face value, this contradicts the model. Previously, we explained this by invoking a “cryptic” source of Wg in *ap* null discs [5–7]. Because the central goal of our present paper is to test the validity of the FF model, we think it important to show the several independent experiments in Fig. 10 that resolve this contradiction by confirming that cryptic Wg explanation.

We note that the experiments in Fig. 10 are also critical to interpretation of the results in Figs. 9, S2 and S3 and are referred to multiple times in the text, as well as the legends of all three Figs.).

We also note that Rev. 3 called attention to the results presented in Fig. 10 as unexpected and of interest.

With respect to the Dpp morphotrap experiments (Fig. 4D), these comprise only a small portion of Fig. 4, which addresses whether wing growth depends on the outward spread of Dpp from source cells via the FF mechanism, so removing them won’t significantly reduce the size of this Figure. On the other hand, we think the comparison between “free” Dpp and morpho-trapped Dpp is a direct and essential test of the importance of the long-range spread of Dpp, and hence is an important experiment that should be shown in the paper proper. The TkvQD experiment is also valuable in showing the critical importance of Dpp input in eliciting the FF response, but it does not directly address the importance of range. We think it valuable to present both experiments, which are mutually reinforcing.

Interpretation of sal expression can be a little difficult given its widespread expression outside of the wing pouch (future experiments might consider using some of the reporters that drive only in the wing pouch).Fig. 5E sal expression in the wing pouch is apparently not dependent on vg (Barrio and De Celis (2004) PNAS)…as well as suggesting it can't be assumed that sal cells also express vg-QE, it also asks the question of whether loss of vg results in complete loss of wing identity

We agree. For this reason, we use Sal expression sparingly, mostly to provide an independent monitor for the receipt of Dpp in prospective wing and pre-wing cells and hence to allow us to assess whether FF wing growth correlates with Dpp input. In the revised manuscript, this is noted on page 12 of the main text (line beginning “*Significantly, both Sal (indicative of . .* .) and page 14 (line beginning “*These clones also induce both Sal and Dll* . . . ), as well as in the legends of Fig. 2 (line beginning “*Note that Sal is expressed at high level* . . . ) and Fig. 3 (line beginning “*The right panel shows the expression of Sal* . . .). These experiments are correlative and provide independent support for the more incisive, subsequent experiments of manipulating Dpp/Tkv input in contexts in which the remaining two inputs, Wg and the FF signal, are not limiting. We also found it useful as a remarkably sensitive indicator of low level QE activation in pre-wing cells in response to ectopic Dpp and WgNrt, confirming that all cells that express Sal in the pre-wing territory also express QE-dependent Vg (Fig. 5E, line beginning “*In both examples, Tub>vgGFP clones* . . . ). As such, we view the Sal data as a valuable addition.

The causal relationship between Vg and Sal expression in pre-wing cells as they begin to be recruited into the wing remains uncertain: in our view it is not resolved by the Barrio and deCelis paper and not critical to any of the interpretations in our paper.

Finally, all of the available data, including our previous ([6] and references therein) and current findings (Fig. 11) indicate that loss of vg results in a complete loss of wing identity.

The premise that Ft is a ligand and Ds is a receptor and is directly activating D downstream is not 'standard' theory in growth control (which has Ds as the ligand, Ft as the receptor which blocks D activity), although there is limited evidence to support this now and this could be pointed out, as some readers will be confused.

Agree: as requested, we now point this out in the legend to Fig. 1 (page 6, lines beginning “*Ft and Ds function as bidirectional ligands and receptors* . . . .), where we explain that Ft and Ds act bidirectionally as ligands and receptors for each other in PCP [8], and that the “on” and “off” states of Vg bias this relationship in the context of wing growth, so that wing cells send a uni-directional Ft-to-Ds signal to pre-wing cells (as established and discussed at length in our previous work [7]).

Relevance to other tissues? What other examples are there of tissue specific transcription factors being regulated by morphogens throughout the whole tissue.

Agree: We now consider the issue of relevance in more depth in the last section of the revised Discussion (page 45, addition of the last paragraph to the two preceding paragraphs which discuss relevance). The simple answer is that we do not, yet, know enough about how morphogens control growth in other tissues, even in other imaginal discs in Drosophila, to address this question. Nevertheless, the control of Drosophila wing growth by Dpp has been a central paradigm for exploring how morphogens govern tissue growth (as we cite, extensively, in the Introduction and Discussion). Hence, as we now argue in the Discussion (page 46, first paragraph), determining how Dpp (and Wg) control wing growth in this context is important in its own right, even if we cannot yet determine how well our present conclusions apply to other contexts.

Is the model relevant only to small tissues such as imaginal discs or very early embryos, what happens when tissues get too big for morphogens to reach all the cells (and the tissue needs to get bigger)?

Agree: this is a fascinating question, going at back to a prescient position paper by Francis Crick in 1971 on the ranges over which morphogens could reasonably be expected to act [9]. However, it is not one that is addressed by the experimental work in the paper, nor is it raised in the many papers about the basic mechanism of growth control by morphogens (and especially Dpp) that we cite. We think discussion of this point falls outside of the purview of the paper.

For what it’s worth, we note that even in the fly wing, there is a late additional stripe of Dpp near the posterior edge of the wing primordium that appears to control the growth of an accessory structure of the wing hinge (the allula;[10]), suggesting that new sources of morphogen may be required to create larger tissues.

When does feed forward end, or does it ever end before pupation?

Agree: this is another fascinating question that is not addressed by the experimental work in the paper. However, it is addressed by an independent body of work (Parker and Struhl, PNAS, in press) in which we provide evidence that FF propagation, and indeed wing size, is limited by morphogen range.

No scale bars, it is difficult to determine how much growth has occurred compared to a real wing

Agree: 50 micron scale bars are now shown in all Figures.

4C cartoon does not match the real image - it does in the other images so was a little confusing to start with, and I don't really see the Dpp-GFP in the N* clone.

Agree: this is a focal plane issue: we have resolved it by providing an image of another clone in which the correspondence is readily visible.

It would have helped to have some of these discs stained for Tsh as well, just to be sure that anywhere vgQE is not active is not simply because a clone abuts cells that can never express it (eg regions labeled x in Fig. 4)

Agree. We have now repeated a few of the key experiments staining for Tsh as well as a second transcription factor Homothorax (which likewise acts in hinge and more proximal territories to distinguish them from the pre-wing/wing domain). The results are as expected and confirm that FF growth only operates within the pre-wing territory, as defined by the absence of Tsh and Hth, but that QE activity is not governed merely by the absence of Tsh and Homothorax. This data is now presented as a Supplemental Figure (Fig. S1).

Vg and Yki are both coactivators….and both bind the same TF, Scalloped…is this just a coincidence?

Agree: as the reviewer implies, it is not a coincidence: the fact that Vg and Yki are coactivators of Sd are indeed relevant to the FF hypothesis. This is because FF input initiates QE-dependent Vg expression — upon which recruitment of pre-wing cells into the wing depends — by inducing Yki activity. Yki then combines with Sd to bind and initiate QE activity in response to Dpp and Wg. As the levels of Vg rise, Vg takes over for Yki and the resulting Vg-Sd complex then auto-regulates QE-dependent Vg expression to sustain the wing state in response to Dpp and Wg.

This is now presented in the legend to the model Figure (Figs. 1E,F); page 5, lines beginning “*Yki is a transcriptional activator that forms a complex with . . .* “

Further…it would have been useful to stain the discs for scalloped expression that are also constitutively expressing vg and which also tkv or arrow mutant clones to see if Wg and Dpp are driving scalloped expression.

Sd expression correlates with Vg expression, being similarly up-regulated wherever Vg is expressed in the wing disc. Indeed, we have evidence that its upregulation appears to be governed solely by Vg. Specifically, Tub>vgGFP clones co-express Sd at high level wherever they are located in the wing disc, even in the prospective hinge and notum territories far from the wing primordium. We infer from this that Sd up-regulation is not normally directly dependent on either Dpp or Wg, but instead only reflects the Dpp, Wg and DSL/Notch dependent expression Vg. Hence, assaying its up-regulation in the experiment in Fig. 11, as the referee suggests, is unlikely to be more informative than assaying QE-dependent Vg expression itself. As the genetics for doing so would be difficult, we have opted not to do this experiment.

We note that the biological role of the Vg-dependent Sd up-regulation in the wing is unknown. In particular, we have found that uniform moderate expression of Sd under the direct control of the Tubα1 promoter fully rescues the development of otherwise sd null animals, indicating that the up-regulation is not critical for normal Vg activity and wing development.

Rev. 3:

The Drosophila wing is an excellent system to study the mechanisms that couple organ identity and growth, a key and long-standing problem in developmental biology which remains poorly understood. During wing development in the fly, the vestigial (vg) gene marks its wing primordium and is necessary for its development. The expression of vg is controlled by a number of enhancers, including early initiating ones (vgBE, vg boundary enhancer) and, at least, a later autoregulatory enhancer, vgQE (vg quadrant enhancer). Work since the 90's has identified major regulatory inputs on vg coupled to patterning events, such as the early dorso-ventral subdivision of the wing disc.

Work by the authors of this manuscript have shown that, after a priming of the prospective wing by early vg expression, the vgQE is necessary and sufficient to provide for enough vg activity to rescue wing development (in apterous mutants. What the contribution of vgQE to the development of the adult wing is still awaits studies where this enhancer is specifically deleted from its genomic locus).

We refer throughout the manuscript to our previous work [5–7], which includes a direct assessment of the independent contributions of QE and BE dependent Vg expression to the specification and growth of the wing (especially Fig. 5 of [5]). The results of these experiments, in which we rescue the vg null condition with transgenes that express Vg under the control of either the QE or BE or both, indicate that the QE is specifically required for the dramatic outward expansion of the wing primordium initially primed by DSL/N dependent activation of the BE. As such, they provided critical early evidence in support of the FF hypothesis at least with regard to Wg.

After defining a series of upstream regulators, that include the (dorso-ventral, DV) activation of Notch and Wg/Wnt signaling, the authors went on to describe a mechanism that explains the coupling of wing growth and wing-fate assignment in the growing tissue. This mechanism is based on a feedforward (FF) loop capable of recruiting pre-wing cells non-autonomously provided that these recruited cells receive Wg signal while, simultaneously, drives their growth through the repression of the Hippo signaling cascade. In all this mechanism, the recruitment of the pre-wing cells rests on
the activation of the vgQE (within the range of Wg's reach) -and therefore, vgQE regulation is critical.

Agree!

In addition to this string of papers, dpp (decapentaplegic, a BMP2 family gene) and its signaling pathway has been shown to play an essential role in wing development. dpp is expressed orthogonally to the DV boundary signaling center and is essential to support the growth of the wing disc and to pattern gene expression along the AP axis. In the present manuscript, the authors ask whether dpp is also required for the FF recruitment and growth of the wing primordium. The authors go a long way, using sophisticated genetic combinations, to show that dpp acts very much like wg -that is, it is required (together with Wg) for the non-autonomous expansion of the vgQE-driven expression of vg.

Agree!

However, as much impressed I am by the genetic analysis and convinced by the FF model, the results presented here were hardly unexpected. It was shown by Kim and co-workers (Nature 1997) that the vgQE is directly regulated by the Dpp signaling pathway by binding of P-Mad (its signal transducer) to (at least) one specific binding site. Therefore, the FF ought to depend on dpp signaling.

With respect, we do not agree.

The prevalent view in the field, as indicated by the extensive primary and review literature that we cite is that Dpp and Wg perform fundamentally different roles in the control of wing development. So much so, that the role of Wg signaling is only rarely considered in the extensive literature on the control of wing growth by Dpp, and there is virtually no mention that the FF mechanism proposed for Wg might apply to Dpp — indeed, the only such citations in the review literature are to our previous speculations [5–7].

The fact that Dpp acts as a morphogen (or at least, its effective diffusion range is limited) would naturally limit the region within the disc that is under Dpp's influence.

Agree, but again, this does not address how the spread of Dpp controls wing growth, and specifically, whether it does so by the FF mechanism or any of the other mechanisms previously proposed.

As we point out in the Introduction and Discussion, there are many other proposals for how Dpp controls wing growth. Aside from “memory” models, all of these would argue that the range of Dpp correlates with wing growth, but they provide very different and diverse explanations. We argue that our evidence that the FF model applies to Dpp provides a new explanation, and one that this reviewer agrees is supported by convincing evidence.

Less critical, but equally important, Dpp signaling has been shown to also modulate the Hippo/yki pathway as a way to stimulate tissue growth in the same direction as the FF recruitment model (Rogulja et al., Dev Cell 2008), so that, in addition to the vg-regulated ft/Ds interface that feeds on the Hippo/yki pathway, dpp signaling might contribute as well.

Agree, but there little if any consideration of how Dpp operates through the Hippo/Yki pathway to control wing growth. Although the Rojulja et al paper [11] implicates the Hippo/Yki pathway, Vg is not mentioned at all in this paper, except for a single sentence at the end of the paper noting the FF hypothesis as an alternative model.

Clones that are defective for the Dpp signaling are known to grow poorly in the wing pouch, so that one would expect that, in rescued wings, the same would hold.

Agree. But this result does not tell us whether this is because such clones cannot express Vg, or because they need Dpp to grow, or both.

In contrast, the experiments in Fig. 11 show that if the failure of such cells to maintain Vg expression is “rescued” by providing exogenous Vg, they still cease growing and drop out of the wing primordium. We view this as a new and important result that establishes a dual requirement for morphogen in both maintaining the wing state and sustaining the growth of wing cells. We now make this point explicitly in the revised Discussion (page 41, paragraph 2).

A less expected finding is that, in the absence of a wing pouch Wg source, the wing surrounding
source of Wg (Wg inner ring, IR) can provide for some Wg and activation of the vgQE. In mutants that lack this expression (spadeflag) the wing hinge is affected, but the adult wing blade develops. Analysis of the spadeflag wings quantitatively may tell whether the IR contribution is at all relevant for wing development if the wg pouch is present.

Agree. In the revised paper, we note that there is little if any detectable effect of the wg[spdflg] mutation on wing size, indicating that Wg derived from inner ring cells normally plays little or no role in the control of wing growth (page 36, paragraph 3).

Frankly, I think that the paper does not constitute a sufficiently large and original improvement of the FF mechanism that the authors have put forward in the past.

Respectfully, we disagree.

In our view it does so by showing that the model applies equally well to Dpp. This consolidates and extends the model, previously based solely on our prior studies of Wg.

In addition, it brings to the fore a new model for Dpp that is distinct from the many previous models.

We are very pleased that this reviewer was impressed by the genetic analysis and convinced by the FF model. We believe that our evidence validating the model and providing a unified mechanism represents a significant advance.

As Rev. 2 concluded: *This is a significant paper and all previous work should be reinterpreted in light of the data presented here*.

Literature cited

1.	Alexandre C, Baena-Lopez A, Vincent J-P. Patterning and growth control by membrane-tethered Wingless. Nature. 2013;505: 180–185. doi:10.1038/nature12879

2.	Morata G, Struhl G. Developmental biology: Tethered wings. Nature. 2014;505: 162–163. doi:10.1038/nature12848

3.	Tian A, Duwadi D, Benchabane H, Ahmed Y. Essential long-range action of Wingless/Wnt in adult intestinal compartmentalization. PLoS Genet. 2019;15: e1008111. doi:10.1371/journal.pgen.1008111

4.	Zecca M, Basler K, Struhl G. Direct and long-range action of a wingless morphogen gradient. 1996;87: 833–844.

5.	Zecca M, Struhl G. Control of Drosophila wing growth by the vestigial quadrant enhancer. Development. 2007;134: 3011–3020. doi:10.1242/dev.006445

6.	Zecca M, Struhl G. Recruitment of cells into the Drosophila wing primordium by a feed-forward circuit of vestigial autoregulation. Development. 2007;134: 3001–3010. doi:10.1242/dev.006411

7.	Zecca M, Struhl G. A feed-forward circuit linking wingless, fat-dachsous signaling, and the warts-hippo pathway to Drosophila wing growth. PLoS Biol. 2010;8: e1000386. doi:10.1371/journal.pbio.1000386

8.	Casal J, Lawrence PA, Struhl G. Two separate molecular systems, Dachsous/Fat and Starry night/Frizzled, act independently to confer planar cell polarity. Development. 2006;133: 4561–4572. doi:10.1242/dev.02641

9.	Crick, F.H.C. Diffusion in Embryogenesis. Nature. 1970;225: 420–422. doi:10.1038/225420a0

10.	Foronda D, Pérez-Garijo A, Martín FA. Dpp of posterior origin patterns the proximal region of the wing. Mech Dev. 2009;126: 99–106. doi:10.1016/j.mod.2008.12.002

11.	Rogulja D, Rauskolb C, Irvine KD. Morphogen control of wing growth through the Fat signaling pathway. Dev Cell. 2008;15: 309–321. doi:10.1016/j.devcel.2008.06.003
